# Research on obstacle avoidance optimization and path planning of autonomous vehicles based on attention mechanism combined with multimodal information decision-making thoughts of robots

**DOI:** 10.3389/fnbot.2023.1269447

**Published:** 2023-09-22

**Authors:** Xuejin Wu, Guangming Wang, Nachuan Shen

**Affiliations:** ^1^College of Transport and Communications, Shanghai Maritime University, Shanghai, China; ^2^School of Management, Wuhan University of Technology, Wuhan, China; ^3^School of Politics and Public Administration, Zhengzhou University, Zhengzhou, China; ^4^Chinese Academy of Fiscal Science, Beijing, China

**Keywords:** attention mechanism, end-to-end architecture, autonomous driving, path planning, multimodal information decision-making for robots, LSTM frontiers

## Abstract

With the development of machine perception and multimodal information decision-making techniques, autonomous driving technology has become a crucial area of advancement in the transportation industry. The optimization of vehicle navigation, path planning, and obstacle avoidance tasks is of paramount importance. In this study, we explore the use of attention mechanisms in a end-to-end architecture for optimizing obstacle avoidance and path planning in autonomous driving vehicles. We position our research within the broader context of robotics, emphasizing the fusion of information and decision-making capabilities. The introduction of attention mechanisms enables vehicles to perceive the environment more accurately by focusing on important information and making informed decisions in complex scenarios. By inputting multimodal information, such as images and LiDAR data, into the attention mechanism module, the system can automatically learn and weigh crucial environmental features, thereby placing greater emphasis on key information during obstacle avoidance decisions. Additionally, we leverage the end-to-end architecture and draw from classical theories and algorithms in the field of robotics to enhance the perception and decision-making abilities of autonomous driving vehicles. Furthermore, we address the optimization of path planning using attention mechanisms. We transform the vehicle's navigation task into a sequential decision-making problem and employ LSTM (Long Short-Term Memory) models to handle dynamic navigation in varying environments. By applying attention mechanisms to weigh key points along the navigation path, the vehicle can flexibly select the optimal route and dynamically adjust it based on real-time conditions. Finally, we conducted extensive experimental evaluations and software experiments on the proposed end-to-end architecture on real road datasets. The method effectively avoids obstacles, adheres to traffic rules, and achieves stable, safe, and efficient autonomous driving in diverse road scenarios. This research provides an effective solution for optimizing obstacle avoidance and path planning in the field of autonomous driving. Moreover, it contributes to the advancement and practical applications of multimodal information fusion in navigation, localization, and human-robot interaction.

## 1. Introduction

Autonomous driving (Huang et al., 2018; Aung et al., 2023; Hu et al., 2023) has become a transformative technology in the transportation industry, offering numerous benefits such as improved road safety, increased traffic efficiency, and enhanced maneuverability. With the growing demand for autonomous vehicles (Bendiab et al., 2023; Min et al., 2023), researchers have been actively exploring various algorithm models to address the challenges related to perception, decision-making, and control in autonomous driving systems. This article aims to provide an overview of the significance and advancements in algorithm models in the field of autonomous driving. Developing effective algorithm models is crucial for the successful implementation of autonomous driving technology. These models enable vehicles to perceive the environment, interpret sensor data (He et al., 2022, 2023), make informed decisions, and execute precise control operations. Understanding and analyzing the different algorithm models used in autonomous driving can help researchers and practitioners gain in-depth insights into the advantages, limitations, and potential areas for improvement in this rapidly evolving field. The following are commonly used models in this field.

Convolutional Neural Networks (CNNs): Used for perception tasks such as object detection and lane detection. They extract meaningful features from sensor data through hierarchical structures, achieving efficient and accurate perception; Recurrent Neural Networks (RNNs), especially LSTM models: Employed for sequence modeling tasks like trajectory and behavior prediction. They capture temporal dependencies to enable dynamic scene prediction and response; Reinforcement Learning (RL): Enables vehicles to learn optimal decision-making by interacting with the environment. Applied in tasks such as motion planning and intersection negotiation, optimizing navigation decisions considering long-term rewards; Bayesian Networks: Provide a probabilistic framework for handling uncertain driving conditions. Utilized in perception fusion and probabilistic environment modeling, achieving robust decision-making in uncertain scenarios; Evolutionary Algorithms: Such as genetic algorithms and particle swarm optimization, utilized for optimization tasks. These algorithms effectively search for optimal solutions in high-dimensional spaces, optimizing parameters, path planning, and system adjustments.

Also in the field of robotics (Cai et al., 2021; Höfer et al., 2021), robot navigation (Vásconez et al., 2023), and path planning (Wu L. et al., 2023) are also important research directions, which have many similarities with the research on obstacle avoidance optimization and path planning of autonomous vehicles in this paper. Autonomous path planning and obstacle avoidance for safe and efficient navigation involve a robot's ability to position and control its movement in space, along with the capability to plan paths and avoid obstacles accordingly. Path planning entails determining the optimal route for a robot to move from a given start to end point within a mapped environment. Obstacle avoidance ensures the robot steers clear of potential obstacles during path planning to ensure operational safety and feasibility. Classic theories and algorithms like A* (Wang X. et al., 2023), Dijkstra's (Ma et al., 2023), and RRT algorithms (Ding et al., 2023) can be drawn upon for guidance in the research, each offering unique characteristics that need to be explored to find effective and feasible shortest paths.

Our approach is based on an end-to-end architecture for autonomous driving systems. The system primarily leverages attention mechanisms and LSTM (Long Short-Term Memory) to optimize obstacle avoidance and path planning tasks. Firstly, we input the multimodal information sensed by the vehicle, such as images and LiDAR data, into the attention mechanism module. The attention mechanism module automatically learns the significant features in the environment (Tang et al., 2021) and weights them accordingly. This enables the system to perceive the environment more accurately and prioritize key information during obstacle avoidance (Ntakolia et al., 2023) and path planning. Secondly, we utilize LSTM models to handle the vehicle's navigation task. LSTM models excel in processing dynamic navigation processes and have memory capabilities to capture dependencies in time-series data (Ragab et al., 2023). By incorporating attention mechanisms to weight key points in the navigation path, the vehicle can flexibly select the optimal path and dynamically adjust it based on real-time conditions. The following are the three contributions of our research:

- Introduction of attention mechanisms: We introduce attention mechanisms into autonomous driving systems. By incorporating attention mechanisms with weighted processing, vehicles can perceive the environment more accurately and prioritize key information. This introduction enhances the robustness and performance of the autonomous driving system, strengthening the vehicle's autonomous obstacle avoidance capabilities.
- Application of LSTM in navigation tasks: We utilize LSTM models to handle the vehicle's navigation task, enabling better navigation in dynamic environments. This LSTM-based navigation approach improves the accuracy and adaptability of vehicle navigation, allowing dynamic adjustments based on real-time conditions.
- Implementation of an end-to-end architecture: Our approach adopts an end-to-end architecture that integrates perception, decision-making, and control into a unified model, building upon the end-to-end architecture of an autonomous driving system. This system heavily relies on attention mechanisms and LSTM for optimizing obstacle avoidance and path planning tasks. Firstly, we input the multimodal information (Wu P. et al., 2023) perceived by the vehicle, such as images and LiDAR data, into the attention mechanism module. The attention mechanism module autonomously learns crucial environmental features and accordingly assigns weights to them. This enables the system to perceive the environment more accurately and prioritize key information during obstacle avoidance and path planning. Secondly, we employ an LSTM model to handle the vehicle's navigation task. The LSTM model is capable of processing navigation processes in dynamic environments and possesses memory capabilities to capture correlations in time-series data. By combining attention mechanisms to weigh key points in the navigation path, the vehicle can flexibly select optimal paths and dynamically adjust them based on real-time conditions.

The logical structure of this article is as follows:

In the second section, we presented related work, described our proposed research methodology, and conducted discussions. The third section introduced the main methods of this paper, such as the attention mechanism, end-to-end architecture, and LSTM. In the fourth section, we discussed the experimental part, including comparisons, ablation experiments, and visualizations (Ezeonu et al., 2023). The fifth section presented the discussion, elaborating on the methodology and recent advancements in the field, highlighting the limitations of our approach, and providing insights into future work. Finally, in the sixth section, we summarized the methodology and provided a conclusive summary.

## 2. Related work

Automatic driving technology, as a transformative technology in the transportation industry, has attracted extensive attention and research. It has many potential benefits, including improving road safety (Jafarzadeh Ghoushchi et al., 2023), enhancing traffic efficiency (Garg and Bouroche, 2023), and increasing mobility. However, achieving reliable automatic driving systems still faces numerous challenges.

Firstly, autonomous vehicles need to accurately perceive and understand complex road environments (Guo et al., 2023). This includes accurate perception and recognition of other vehicles, pedestrians, traffic signals, road signs, and geometric structures. Accurate environmental perception forms the foundation for making informed decisions in autonomous driving systems. However, this method may be affected by environmental changes, such as adverse weather conditions, insufficient light, or sensor failures. In these situations, the perception system may not be able to obtain sufficiently accurate information, leading to the system making incorrect decisions. In addition, accurate perception and recognition require highly complex algorithms and sensors, which may lead to increased system costs and deployment complexity.

Secondly, autonomous driving systems require efficient decision-making capabilities. They need to make rapid and accurate decisions based on the perceived environmental information, such as obstacle avoidance, path planning, and traffic participation. This is crucial for ensuring safe and efficient vehicle operation in complex traffic environments.

Additionally, precise control capabilities (Chotikunnan and Pititheeraphab, 2023) are necessary for autonomous driving systems to achieve accurate vehicle maneuvering. This includes controlling vehicle acceleration, braking, steering, and precise control of vehicle power systems and braking systems. However, in practical applications, achieving precise control may be influenced by multiple factors. For example, changes in road conditions, background traffic conditions, and unforeseeable events can all interfere with precise control. This may result in the control system needing to adjust in real-time to adapt to changing situations, but the system may not be able to provide optimal response in all situations, and sensors may also have delays and noise.

In this context, researchers and practitioners have been actively exploring various algorithm models and technological methods to address the challenges related to perception, decision-making, and control in autonomous driving systems. They aim to develop more accurate, efficient, and reliable algorithm models to enhance the performance and reliability of autonomous driving systems. These research efforts are aimed at promoting the development of automatic driving technology and providing better solutions for practical application scenarios.

In this regard, perception forms the foundation of autonomous driving systems and involves accurate perception and recognition of road environments, obstacles, and traffic signs. In perception research, numerous theoretical and experimental research findings have been achieved. In literature (Zhang et al., 2023), researchers have utilized various sensors (Liu et al., 2021) such as cameras, lidar, etc., for environment perception and obstacle detection. CNN have been widely applied for object detection and lane detection tasks, enabling accurate and efficient perception by extracting meaningful features from sensor data. However, despite CNN's excellent performance in object detection and lane detection, there are still some limitations and drawbacks. For example, in dealing with complex situations such as occlusion, changes in lighting, and different perspectives, it may be affected. The quality of sensor data and changes in environmental conditions may make it difficult for CNN to accurately identify obstacles or lane lines; Its demand for a large amount of annotated data may limit its generalization ability beyond specific scenarios or datasets. Without sufficient diversity data for training, CNN may not perform well in various complex environments.

Decision-making (Wang F.-Y. et al., 2023) is crucial in autonomous driving systems, where decisions need to be made based on the perceived environmental information, such as obstacle avoidance, path planning, and traffic participation. In decision-making research, various decision-making algorithms and models have been developed. RL algorithms have been widely applied, enabling vehicles to learn optimal decision-making strategies through interactions with the environment, such as motion planning, lane changing, and negotiation at intersections. However, in decision-making research, the RL algorithm may require a large amount of training data and time to achieve good performance. In complex traffic environments and uncertain road conditions, a large number of experiments and interactions are required to adjust and optimize decision strategies, which may limit the practical application of the algorithm.

Moreover, other research areas have received significant attention and application in the field of autonomous driving. Sensor fusion techniques have been employed to integrate information from multiple sensors (Shao et al., 2023a), improving the reliability and accuracy of perception. Path planning algorithms aim to find optimal driving paths, considering road conditions, traffic situations, and vehicle capabilities, enabling efficient and safe vehicle operation in complex traffic environments.

For example, in the robotic system that integrates perception and decision-making, the perception module, and decision-making module play an important role (Xu W. et al., 2023), including visual sensors, lidar, radar, inertial measurement units, etc. These sensors are capable of acquiring multimodal information about the vehicle's surroundings, such as data such as images, point clouds, and distances. By processing and analyzing these data, the perception module can extract key environmental features, such as roads, vehicles, pedestrians and obstacles, and classify, locate and track them. The decision-making module is responsible for making intelligent decisions based on the information provided by the perception module. In literature (Black et al., 2023), the controller converts the path generated by the planner into specific vehicle control instructions, and controls parameters such as the speed, steering and acceleration of the vehicle. Compared with traditional robotic systems, our method has obvious advantages in the fusion of perception and decision-making. First, the method in this paper adopts an end-to-end architecture, which integrates perception and decision-making tasks into one model, avoiding the information transfer and alignment problems between perception and decision-making in traditional systems, and making the whole system more compact and efficient. Secondly, the method in this paper introduces an attention mechanism, which enables the vehicle to pay more attention to important environmental features and obstacles, improving the accuracy of perception and the robustness of decision-making. In terms of dynamic environment perception for robots, this is a key problem in solving the perception and decision-making of autonomous vehicles in complex and dynamic environments. In such an environment, vehicles need to be able to accurately perceive and track moving objects and obstacles in order to make timely decisions and plan driving paths. In our approach, these dynamic environment perception techniques can be combined to improve the perception and decision-making capabilities of autonomous vehicles.

To sum up, with the rapid development of deep learning (Zhang M. et al., 2022; Zhang Y.-H. et al., 2022), a large number of theoretical, experimental and applied researches have been carried out in the field of automatic driving, which provides valuable theoretical basis and technical support for the development and application of automatic driving systems. However, there may still be the following research gaps: comparison of multimodal information fusion methods; Selection of different end-to-end architectures; The application of classic algorithms in the field of robotics; Traffic behavior modeling; Human machine interaction and driver behavior prediction; Adaptability to urban and non urban environments; Diversity of experimental evaluations; Actual deployment and application cases, etc.

## 3. Method

**Figure 1:**
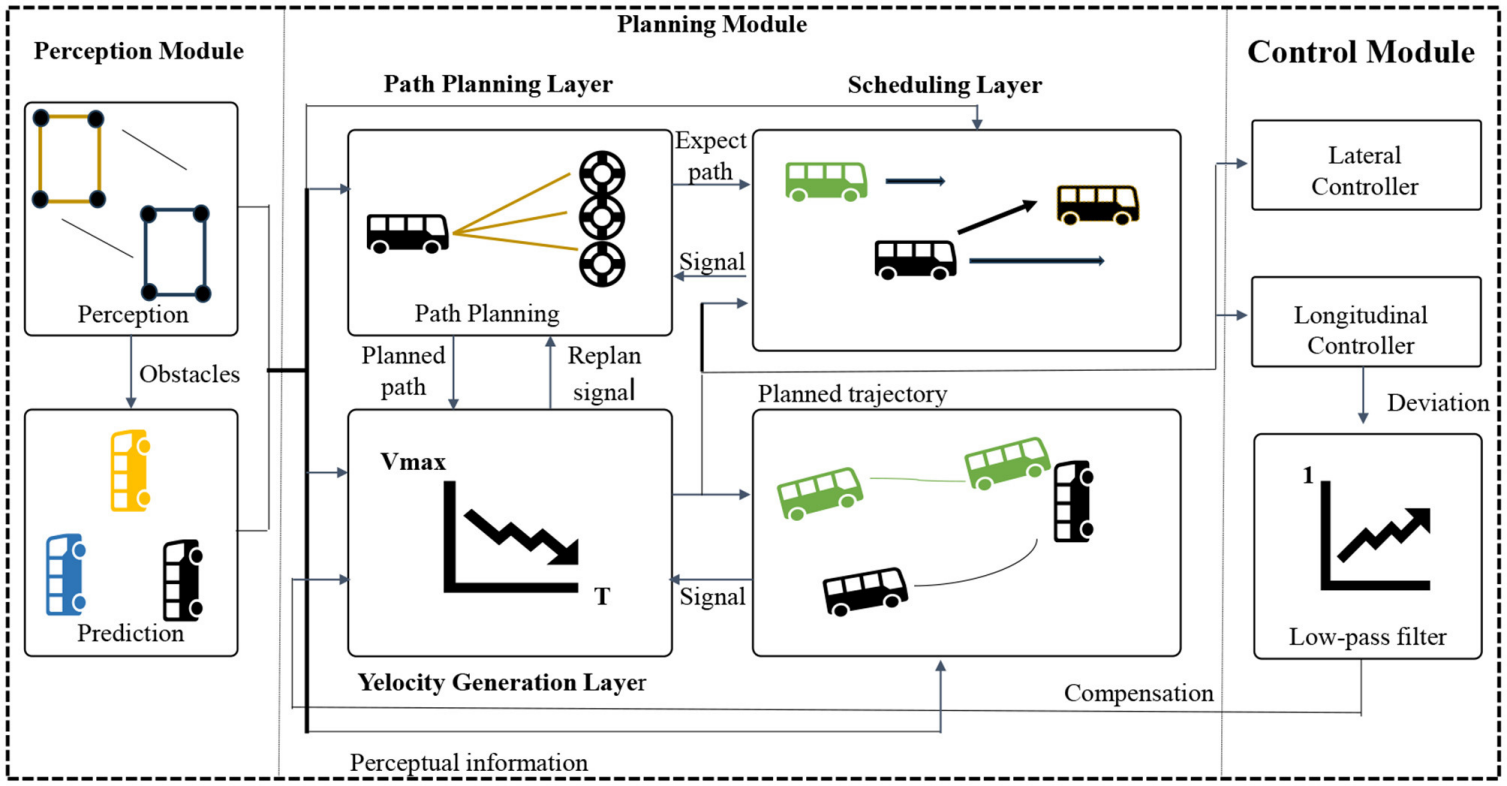
Overall algorithm flowchart.

The overall algorithm flow chart of this paper is shown in Figure 1.

### 3.1. Attention mechanism

The structure of an attention mechanism (Zhao et al., 2023) can be divided into three main components: Query, Key, and Value. These components work together to enable the model to weight the values based on the relationship between the query and the keys.

The query is a vector that specifies the information the attention mechanism should focus on. It can be an internal representation of the model or an input from the external context. The query is used to compute the similarity between the query and each key, determining the weights assigned to each value.

The key is also a vector and represents the features or contextual information in the attention mechanism. The calculation of similarity between the query and keys determines the weight for each corresponding value.

The value is a set of vectors corresponding to the keys, storing the actual information that the attention mechanism processes. The values can be internal representations of the model or inputs from the external context. The attention mechanism combines the values based on their weights, resulting in a weighted sum that represents the final output. By computing the similarity between the query and each key, and transforming the similarities into weights, the attention mechanism focuses on the values that are most relevant to the query.

**Figure 2:**
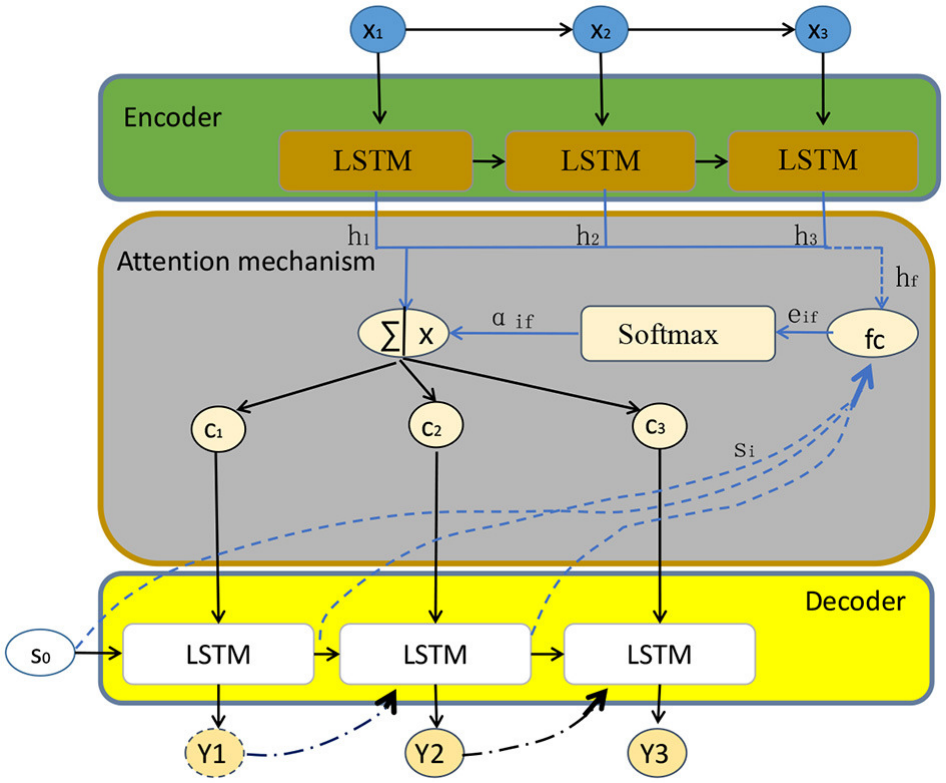
Attention mechanism model diagram.

The model diagram of the attention mechanism is shown in Figure 2.

The basic principle of the attention mechanism involves calculating the similarity between the query (Q) and the keys (K), and converting the similarity into weights to combine the values (V). Here are the equations that need to be derived:


(1) $$\begin{array}{l} \text{Similarity}=\text{Q}\cdot {\text{K}}^{T} \end{array}$$

(2) $$\begin{array}{l} \text{Weights}=\text{Softmax}\;\left(\text{Similarity}\right) \end{array}$$

(3) $$\begin{array}{l} \text{Output}=\text{Weights}\cdot \text{V} \end{array}$$

In these equations, Q represents the query vector, K represents the key vector, and V represents the value vector. The similarity is computed by taking the dot product between the query and the transpose of the key. The softmax function is applied to the similarity vector to convert it into a probability distribution, ensuring that the weights sum up to 1. Finally, the output is obtained by multiplying the weights with the values, resulting in a weighted sum of the values.

In addition, we conducted weight fusion on multimodal information, which is explained as follows:


(4) $$\begin{array}{l} W_{fusion}=\overset{N}{\underset{i=1}{\sum }}w_{i}\cdot M_{i} \end{array}$$

Among them, *W*_*fusion*_ represents the fused multimodal information, N represents the quantity of multimodal information, *W*_*i*_ represents the weight of the i-th type of information, and *M*_*i*_ represents the raw data of the i-th type of information. The weight calculation of the attention mechanism is as follows:


(5) $$\begin{array}{l} \alpha_{i}=\frac{exp\left(e_{i}\right)}{\sum_{j=1}^{N}exp\left(e_{j}\right)} \end{array}$$

(6) $$\begin{array}{l} e_{i}=f\left(W_{att}\cdot x_{i}\right) \end{array}$$

α_*i*_ represents the attention weight of the i-th information, *x*_*i*_ represents the feature representation of the i-th information, *W*_*att*_ represents the weight matrix of the attention mechanism, and f represents the activation function. The attention weight in path planning is represented as follows:


(7) $$\begin{array}{l} \beta_{i}=\frac{exp\left(d_{i}\right)}{\sum_{j=1}^{M}exp\left(d_{j}\right)} \end{array}$$

(8) $$\begin{array}{l} d_{i}=g\left(U_{att}\cdot h_{i}\right) \end{array}$$

β_*i*_ represents the attention weight of the i-th point in the navigation path, M represents the number of path points, *h*_*i*_ represents the feature representation of the i-th point, *U*_*att*_ represents the weight matrix of path attention, and g represents the activation function.

The attention mechanism allows the model to selectively focus on relevant information based on the relationship between the query and the keys. It provides a flexible way to process different parts of input sequences, improving the performance, and representation capabilities of the model.

### 3.2. End-to-end architecture

**Figure 3:**
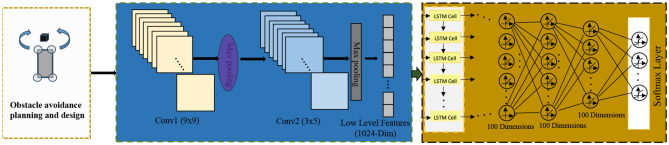
End-to-end architecture diagram.

The end-to-end architecture (Shao et al., 2023b) is a method that integrates multiple modules or components into a unified model. It allows input data to flow directly through different parts of the model, leading to the final output without the need for manual design of intermediate steps. In the field of autonomous driving, the end-to-end architecture is widely used in the design and implementation of the entire autonomous driving system. Its architecture diagram is shown in Figure 3.

It mainly consists of the following components:

Perception Module: The perception module is responsible for receiving raw data from sensors (such as images, LiDAR data, etc.) and transforming it into a form that the model can understand and process. The perception module typically includes sub-modules such as image processing, feature extraction, and object detection, which extract meaningful features and information from the input data.

Decision Module: The decision module receives the features and information extracted by the perception module and makes appropriate decisions based on the current environment and task requirements. The decision module can be based on various algorithms and models, such as reinforcement learning, rule-based engines, or optimization methods, to achieve intelligent decision-making of the autonomous driving system in different scenarios.

Control Module: The control module is responsible for converting the output instructions from the decision module into actual control signals that control the vehicle's acceleration, braking, steering, and other operations. The control module may include sub-modules for vehicle power systems, braking systems, steering systems, etc., to ensure accurate control of the vehicle according to the instructions from the decision module.

In the end-to-end architecture, the following equation can be used to represent its basic principle:


(9) $$\begin{array}{l} \text{Output}=f\left(\text{Input}\right) \end{array}$$

Here, the input represents the input data, and the output represents the final output of the model. The function f represents the entire end-to-end model, which directly maps the input data to the output result. This function f can be a complex nonlinear function composed of the perception module, decision module, and control module.

In this context, combined with end-to-end architecture, multimodal path planning decisions can be expressed as:


(10) $$\begin{array}{l} P_{path}=\arg\underset{P_{i}}{\max}\left(\overset{N_{c}}{\underset{i=1}{\sum }}\gamma_{i}\cdot S_{i}\left(P_{i}\right)\right) \end{array}$$

*P*_*path*_ represents the optimal path planning, *P*_*i*_ represents the i-th path planning candidate, *N*_*c*_ represents the number of path planning candidates, γ_*i*_ represents the weight of path planning candidates, and *S*_*i*_(*P*_*i*_) represents the score of path planning candidate *P*_*i*_. Decision systems based on multimodal information can be represented as:


(11) $$\begin{array}{l} A_{decision}=\arg\underset{A}{\max}\left(\overset{M}{\underset{i=1}{\sum }}\delta_{i}\cdot F_{i}\left(A\right)\right) \end{array}$$

Among them, *A*_*decision*_ represents the optimal decision, *A* represents the set of decision candidates, *M* represents the number of decision candidates, δ_*i*_ represents the weight of decision candidates, and *F*_*i*_(*A*) represents the evaluation function of decision candidate *A*. The path planning decision for multimodal information fusion paths can be expressed as:


(12) $$\begin{array}{l} P_{final}=\arg\underset{P_{path}}{\max}\left(\overset{M_{c}}{\underset{i=1}{\sum }}{\text{λ}}_{i}\cdot G_{i}\left(P_{path}\right)\right) \end{array}$$

Among them, *P*_*final*_ represents the final path planning decision, *P*_*path*_ represents the path planning decision candidate, *M*_*c*_ represents the number of path planning decision candidates, λ_*i*_ represents the weight of path planning decision candidates, and *G*_*i*_(*P*_*path*_) represents the score of path planning decision candidate *P*_*path*_.

In the end-to-end architecture, these components are combined into a unified model, and data can flow directly between different components. The data goes through the perception module from the input, then processed by the decision module, and finally, the control module outputs control commands. This end-to-end design approach eliminates the intermediate steps and manual feature engineering in traditional separate designs, making the system more simplified, efficient, and easy to debug. Its advantage lies in the ability to automatically learn the optimal representation and decision strategies from raw data, while reducing information loss and error propagation in manual design steps. However, the end-to-end architecture also faces challenges such as the need for large amounts of data and computational resources, interpretability, and robustness, which require further research and improvement.

### 3.3. LSTM

LSTM (Qin et al., 2023) is a special architecture used in RNNs for processing sequential data. It excels in addressing the issue of long-term dependencies that traditional RNNs face and has been widely applied in tasks such as natural language processing, speech recognition, and time series prediction.

**Figure 4:**
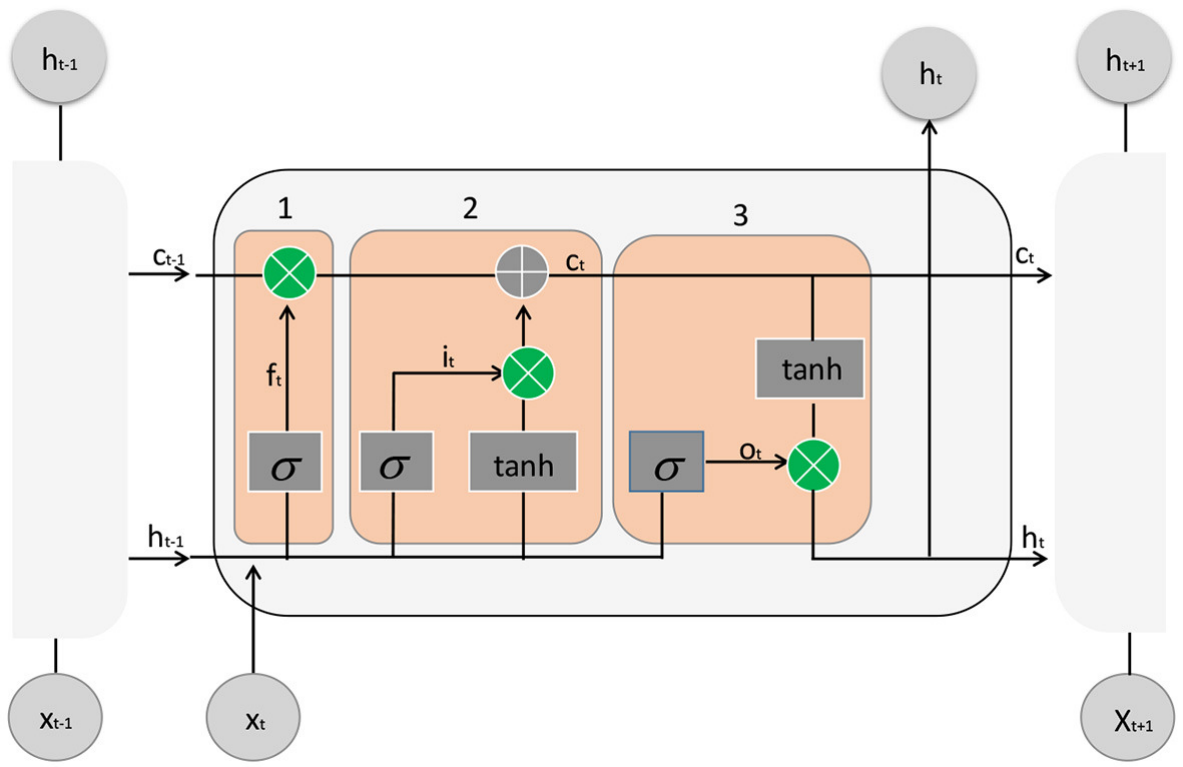
The structure diagram of LSTM usually consists of multiple repeated units, each containing three gating structures: forget gate, input gate, and output gate.

The LSTM architecture consists of a cell state and three gate units: the input gate, forget gate, and output gate. These gate units dynamically control the flow of information within the cell state and determine which parts of the current input should be remembered or forgotten. The LSTM model diagram is shown in Figure 4.

Here is a detailed description of the LSTM architecture:

Input Gate: The input gate decides which parts of the current input information should be included in the cell state update. It calculates a value between 0 and 1, denoted as *i*_*t*_, based on the current input (*x*_*t*_) and the previous hidden state (*h*_*t*−1_), representing the importance of the corresponding positions' information.


(13) $$\begin{array}{l} i_{t}=\sigma \left(W_{xi}x_{t}+W_{hi}h_{t-1}+b_{i}\right) \end{array}$$

Here, *W*_*xi*_ and *W*_*hi*_ are weight matrices, *b*_*i*_ is a bias vector, and σ represents the sigmoid function.

Forget Gate: The forget gate determines which old memories should be forgotten. It calculates a value between 0 and 1, denoted as *f*_*t*_, based on the current input (*x*_*t*_) and the previous hidden state (*h*_*t*−1_), indicating the retention level of the corresponding positions' old memories.


(14) $$\begin{array}{l} f_{t}=\sigma \left(W_{xf}x_{t}+W_{hf}h_{t-1}+b_{f}\right) \end{array}$$

Cell State Update: The cell state update calculates a candidate new cell state ($\tilde{C}t$) to replace the old cell state (*Ct*−1). It is based on the current input (*x*_*t*_) and the previous hidden state (*h*_*t*−1_).


(15) $$\begin{array}{l} \tilde{C}t=\tanh\left(Wxcx_{t}+W_{hc}h_{t-1}+b_{c}\right) \end{array}$$

Here, tanh represents the hyperbolic tangent function.

Cell State Update: The cell state is updated by combining the old cell state (*C*_*t*−1_) and the new candidate cell state ($\tilde{C}t$) using the forget gate (*f*_*t*_) to control the forgetting of old memories and the input gate (*i*_*t*_) to control the update of new memories.


(16) $$\begin{array}{l} C_{t}=f_{t}⊙Ct-1+i_{t}⊙{\tilde{C}}_{t} \end{array}$$

Here, ⊙ represents element-wise multiplication.

Output Gate: The output gate determines which information should be included in the current hidden state (*h*_*t*_). It calculates a value between 0 and 1, denoted as *o*_*t*_, based on the current input (*x*_*t*_) and the previous hidden state (*h*_*t*−1_), indicating the degree of output for the corresponding positions' information.


(17) $$\begin{array}{l} o_{t}=\sigma \left(W_{xo}x_{t}+W_{ho}h_{t-1}+b_{o}\right) \end{array}$$

Hidden State Update: The hidden state is updated by activating the cell state (*C*_*t*_) with a hyperbolic tangent function and using the output gate (*o*_*t*_) to control the output information.


(18) $$\begin{array}{l} h_{t}=o_{t}⊙\tanh\left(C_{t}\right) \end{array}$$

In an LSTM, these gate units dynamically compute and adjust the weights of inputs and outputs, allowing the model to selectively retain and update information, thereby better capturing long-term dependencies in sequential data. This architecture design enhances the performance and representation power of the model when dealing with sequential data.

## 4. Experiment

**Figure 5:**
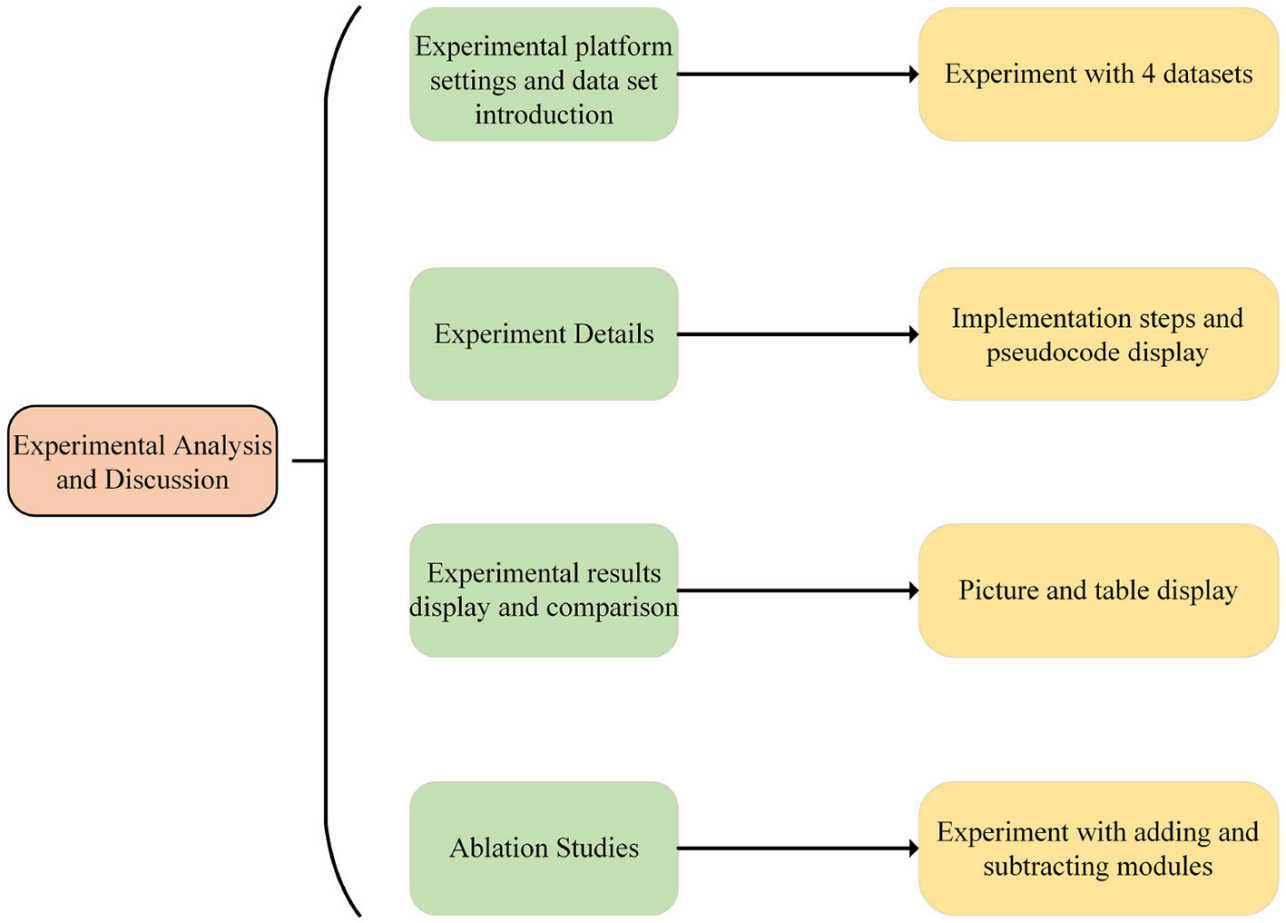
Experiment flow chart.

The experimental flow chart of this paper is shown in Figure 5.

### 4.1. Experimental platform settings and data set introduction

The experiment was conducted on a Windows 10 operating system with an Intel processor. PyTorch was used as the primary deep learning framework, providing a rich set of tools and interfaces for model construction, training, and evaluation. The GPU used was an NVIDIA GeForce RTX 2080 Ti, which offers powerful computational capabilities and ample memory capacity to accelerate model training and inference processes. To leverage the GPU's computing power, CUDA and cuDNN libraries were installed to enable parallel computing and accelerate the training and inference of deep learning models. The initial learning rate for training was set to 0.001, and the coding environment used was PyCharm.

Waymo open dataset: It contains a rich variety of sensor data, including high-resolution lidar data, high-definition camera images, GPS and inertial measurement unit (IMU) data, etc. The dataset covers a variety of scenarios and driving situations, including driving on urban roads, highways, and severe weather conditions. The dataset also provides precise vehicle calibration information and vehicle motion trajectories, as well as rich semantic annotations, such as lane lines, traffic lights, pedestrians, and other vehicles, etc.

The apolloscape open dataset:It covers driving scenarios in multiple cities and under different environmental conditions, such as city streets, highways, parking lots, etc. The dataset provides high-resolution lidar data, panoramic images, semantic segmentation labels, vehicle behavior annotations, and more. These data can help researchers understand and simulate real-world autonomous driving scenarios, and promote the performance improvement of algorithms and systems.

The kitti vision benchmark suite:It is based on actual collected urban street scenes, including different driving scenarios such as urban roads, highways and rural roads. The dataset provides data from multiple sensors, including lidar, camera, GPS, and inertial measurement unit (IMU). Lidar data provides point cloud information, camera data includes RGB images and grayscale images, and GPS and IMU data provide positioning and attitude information. These data can simulate the real driving environment and provide rich input for the research and evaluation of the algorithm.

The cityscapes dataset: It is based on real street scenes of German and other European cities, including city streets, intersections, buildings, pedestrians, vehicles, and many other objects. The dataset provides high-resolution RGB images and corresponding pixel-level annotations. The resolution of the images is 1,024 × 2,048, and the annotations include 33 different categories, such as roads, sidewalks, vehicles, traffic lights, etc. These images and annotations can simulate real urban environments and provide meaningful input for algorithm research and evaluation.

### 4.2. Experiment details

The first is the establishment of the vehicle model, including kinematic constraints, two-point boundary constraints, and collision avoidance constraints. Its model can be expressed as:


(19) $$\begin{array}{l} \dot{x}=v\cdot \text{cos}\left(\theta \right) \end{array}$$

(20) $$\begin{array}{l} \dot{y}=v\cdot \text{sin}\left(\theta \right) \end{array}$$

(21) $$\begin{array}{l} \dot{\theta }=\frac{v}{L}\cdot \text{tan}\left(\delta \right) \end{array}$$

**Figure 6:**
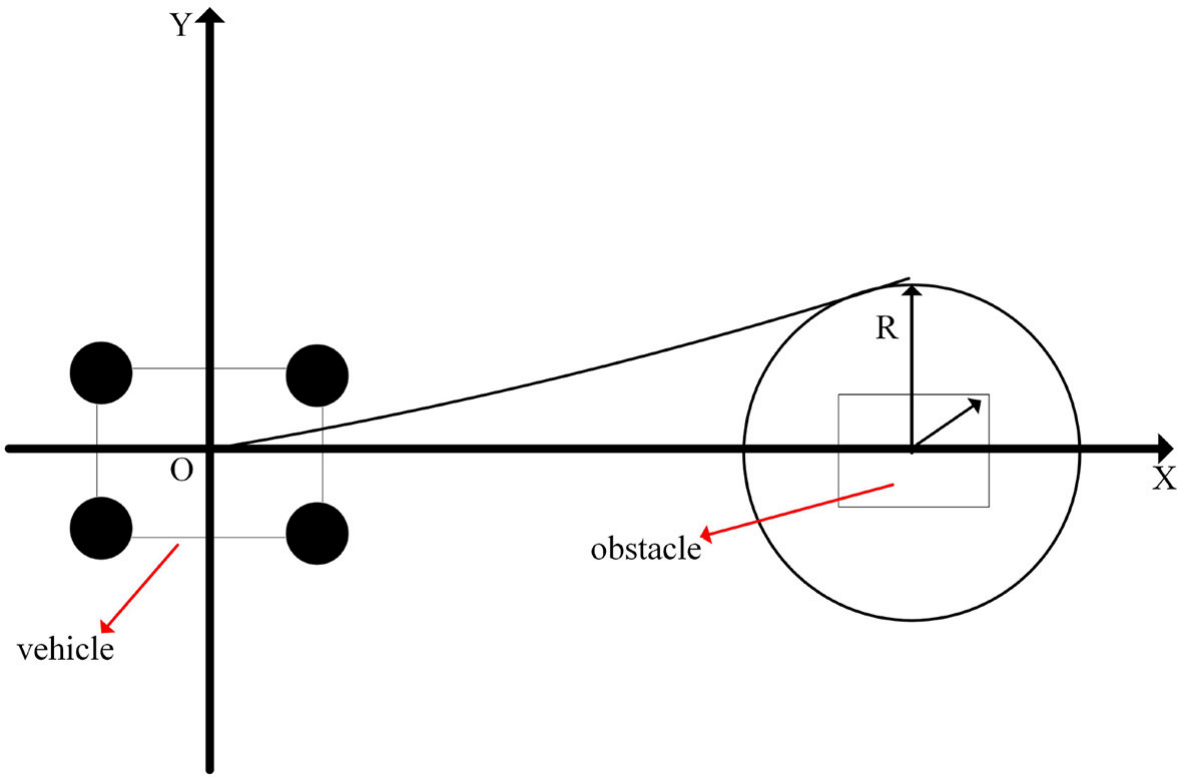
Schematic diagram of obstacle avoidance path planning.

The effect of its obstacle avoidance path planning is shown in Figure 6.

That is, it is assumed that the vehicle is a mass point, and the rotation and lateral motion of the vehicle are ignored. Among them, *ẋ* and *ẏ* represent the velocity components of the vehicle in the x and y directions; *v* represents the linear velocity of the vehicle; θ represents the heading angle of the vehicle; $\overset{•}{\theta }$ represents the angular velocity of the vehicle; *L* represents the wheelbase of the vehicle (the distance from the center of the front wheel to the center of the rear wheel); δ represents the steering angle of the vehicle. This model describes the law of motion of the vehicle during straight driving and turning.

Set the vehicle length *L*, vehicle width *W*, vehicle height *H*, vehicle total mass *m*, obstacle detection range detection radius *r*_*d*_, safety distance *d*_*s*_, obstacle avoidance path Generate distance *d*_*p*_. Among them, the distance formula between the obstacle and the vehicle is:


(22) $$\begin{array}{l} d=\sqrt{{\left(x-x_{o}\right)}^{2}+{\left(y-y_{o}\right)}^{2}} \end{array}$$

We simplify the vehicle to a point mass model, the vehicle position coordinates (*x, y*), and the vehicle orientation angle is θ. In terms of obstacle detection, use the on-board sensor to obtain the surrounding environment information, the detected obstacle position (*x*_*o*_, *y*_*o*_) and size information, and calculate the vehicle and obstacle based on the detected obstacle position and vehicle pose information The distance *d* and the relative angle α of. According to *d*, α, and other parameters, determine whether there is a potential safety hazard, and plan the optimal obstacle avoidance path.

The formula for the relative angle of obstacles is:


(23) $$\begin{array}{l} \alpha =\arctan\left(\frac{y_{o}-y}{x_{o}-x}\right) \end{array}$$

The formula for the tangent distance from the vehicle to the obstacle is:


(24) $$\begin{array}{l} d_{t}=d-r-\frac{L}{2} \end{array}$$

In the experimental setting of this paper, the vehicle needs to drive from the centerline of one side of the road to the other side of the road to avoid obstacles ahead. The path planning process is: when an obstacle is detected, calculate the distance *d* from the vehicle to the obstacle and the relative angle α of the obstacle. And judge whether there is a potential safety hazard. If *d*<*r*_*d*_, there is a potential safety hazard, and obstacle avoidance planning is required. Therefore, we need to plan the optimal obstacle avoidance path: first calculate the tangent distance *d*_*t*_ between the vehicle and the obstacle to ensure that the vehicle has enough distance to brake and not collide with the obstacle; secondly, according to *d*_*t*_ and the size of the vehicle, Determine the position (*x*_*new*_, *y*_*new*_) of the other side of the road where the vehicle should drive to; then according to (*x*_*new*_, *y*_*new*_) and the current position of the vehicle (*x, y*), planning the obstacle avoidance path; after the obstacle avoidance path is generated, the vehicle drives along the path until it avoids the obstacle, and after the obstacle avoidance, the vehicle returns to the original lane to continue driving.

The formula for the position of the vehicle traveling to the other side of the road is:


(25) $$\begin{array}{l} x_{\text{new}}=x_{o}-d_{t}\cos\alpha \end{array}$$

(26) $$\begin{array}{l} y_{\text{new}}=y_{o}-d_{t}\sin\alpha \end{array}$$

Next, in terms of dynamic obstacle area division, the position and speed of dynamic obstacles (other moving vehicles) are monitored and updated based on the vehicle's perception information. According to the relationship between the position and speed of dynamic obstacles and the current position of the vehicle, the surrounding area is divided into different danger levels, such as short-distance danger zone, middle-distance danger zone and long-distance danger zone; in the static obstacle area division: static obstacle The position and shape of objects (such as roadblocks, buildings, etc.) can be detected and measured by lidar or camera sensors. According to the relative position and distance of obstacles and vehicles, the surrounding area is divided into static obstacle areas.

The planning path for avoiding the dynamic obstacle vehicle is: target positioning: obtain the position and speed information of the dynamic obstacle vehicle through the vehicle perception system; path generation: use the path planning algorithm to generate an avoidance path according to the position and speed of the dynamic obstacle vehicle. The path should be as far away from the dynamic obstacle car as possible, and ensure the safety of the vehicle; Path selection: Among the generated avoidance paths, select the optimal path, taking into account the length of the path, safety and comfort of the vehicle. The optimal path should have enough distance to ensure that the vehicle safely avoids the dynamic obstacle car; path tracking: according to the selected path, the vehicle control algorithm is used to guide the vehicle to the target path. The control algorithm can use feedback control or model predictive control methods to maintain the correct position and speed of the vehicle on the planned path. The planning path for avoiding static obstacle vehicles is: Obstacle detection: use laser radar or camera sensor to detect the position and shape information of static obstacle vehicles; Obstacle prediction: By analyzing the movement mode of obstacles and the movement state of vehicles, predict Future position and path; path generation: According to the position and forecast information of static obstacles, a path planning algorithm is used to generate a safe avoidance path. The path should bypass static obstacles to ensure the safe passage of the vehicle; Path selection: Among the generated avoidance paths, select the optimal path, taking into account the length of the path, safety, and comfort of the vehicle. The optimal path should maintain a sufficient distance to ensure that the vehicle safely bypasses static obstacles; path tracking: Based on the selected path, the vehicle control algorithm is used to guide the vehicle to the target path. The control algorithm can use feedback control or model predictive control methods to maintain the correct position and speed of the vehicle on the planned path. These steps can achieve intelligent obstacle avoidance for autonomous vehicles through the integration of vehicle perception systems, path planning algorithms, and control algorithms Function.

The pseudocode of its path planning is shown in Algorithm 1.

**Algorithm 1:**
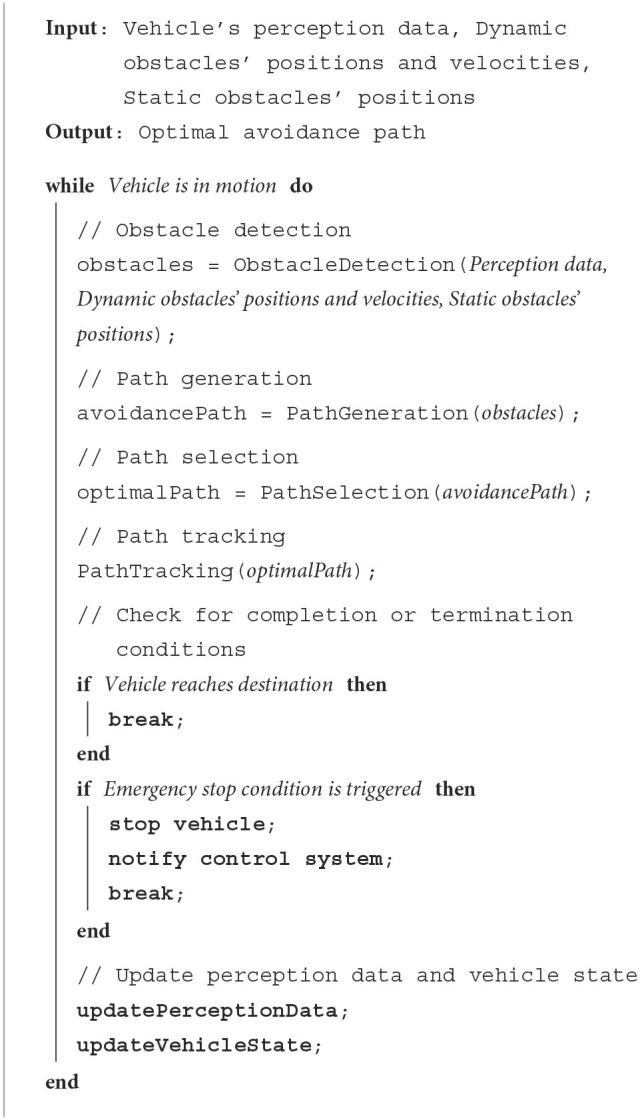
Obstacle avoidance path planning.

### 4.3. Experimental results display and comparison

We conducted experiments on four datasets and compared our method with state-of-the-art approaches in recent years. The results demonstrate that our method exhibits promising performance. As shown in Table 1.

**Table 1:** Experimental comparison between Waymo open dataset and the apolloscape open dataset, where “LE,” “ODA,” “OASR,” and “EE,” respectively represent Localization Error, Object Detection Accuracy, Obstacle Avoidance Success Rate, and Energy Efficiency.

**Method**	**Datasets**							
	**Waymo open dataset (Sun et al.**, **2020****)**				**The apolloscape open dataset (Huang et al.**, **2019****)**			
	**LE (m)**	**ODA (%)**	**OASR (%)**	**EE (kWh/km)**	**LE (m)**	**ODA (%)**	**OASR (%)**	**EE (kWh/km)**
Xue et al. (2023)	3.49	85.16	92.45	0.31	2.91	78.69	95.81	0.16
Hao et al. (2023)	4.24	77.53	95.31	0.27	1.66	72.83	94.04	0.24
Taghavifar et al. (2023)	3.66	88.79	92.86	0.20	3.36	82.95	95.19	0.15
Lin et al. (2023)	1.21	77.86	91.48	0.11	4.57	81.77	94.96	0.43
Tan et al. (2023)	3.06	82.38	91.76	0.34	4.28	70.28	92.94	0.41
Xu L. et al. (2023)	1.13	82.94	94.29	0.44	4.62	72.02	95.61	0.16
Ours	1.02	93.33	96.97	0.10	1.23	87.79	95.89	0.11

We compared and evaluated four metrics, namely LE (Localization Error), ODA (Object Detection Accuracy), OASR (Obstacle Avoidance Success Rate), and EE (Energy Efficiency), on the Waymo open dataset. Regarding the LE metric, we compared the localization errors of different methods, and the results showed that Method 2 achieved an LE value of 3.49, while our method achieved an LE value of 1.02. The closest performance to our method was achieved by Method 6, with an LE value of 1.13. This indicates that our method outperforms others in terms of the LE metric, further validating the advantage of our method in terms of localization accuracy. For the ODA metric, our method achieved an accuracy rate of 93.33%, surpassing other methods such as Method 1, Method 2, Method 3, Method 4, Method 5, and Method 6. This demonstrates that our method exhibits higher accuracy in object detection and localization. In terms of the OASR metric, our method achieved a success rate of 96.97%, outperforming other methods. The OASR values for Method 1, Method 2, Method 3, Method 4, Method 5, and Method 6 were 85.16, 77.53, 88.79, 77.86, 82.38, and 82.94%, respectively, which are significantly lower compared to our method. Regarding the EE metric, our method achieved an energy efficiency of 0.10 kWh/km, demonstrating more efficient utilization of energy resources compared to other methods. The EE values for Method 1, Method 2, Method 3, Method 4, Method 5, and Method 6 were 0.31, 0.27, 0.20, 0.11, 0.34, and 0.44 kWh/km, respectively, which are noticeably lower than our method. Taking into account the ODA, OASR, and EE metrics, our method outperforms the other six methods in the Waymo open dataset. Whether it's object detection accuracy, obstacle avoidance capability, or energy utilization efficiency, our method demonstrates superior performance and superiority. In the ApolloScape open dataset, we first focus on the LE metric.

**Figure 7:**
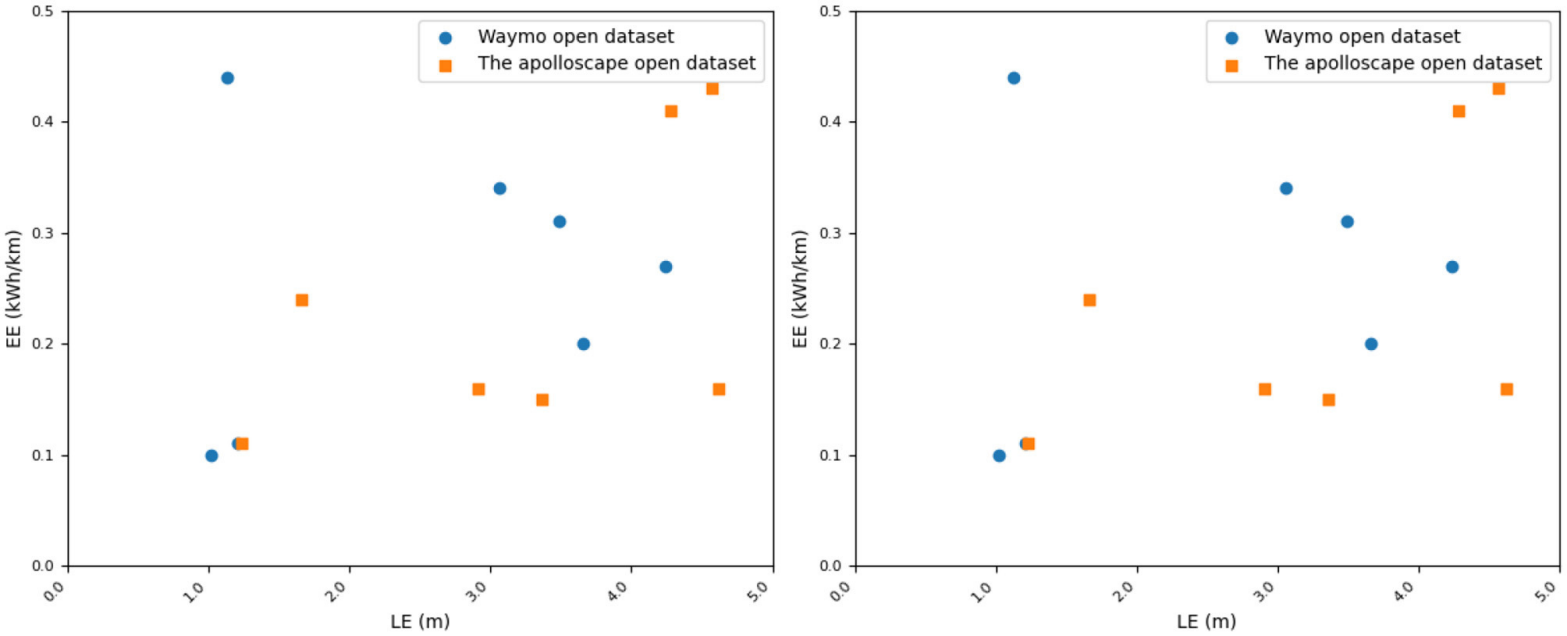
Comparison of Waymo open dataset and the apolloscape open dataset in terms of indicators.

**Figure 8:**
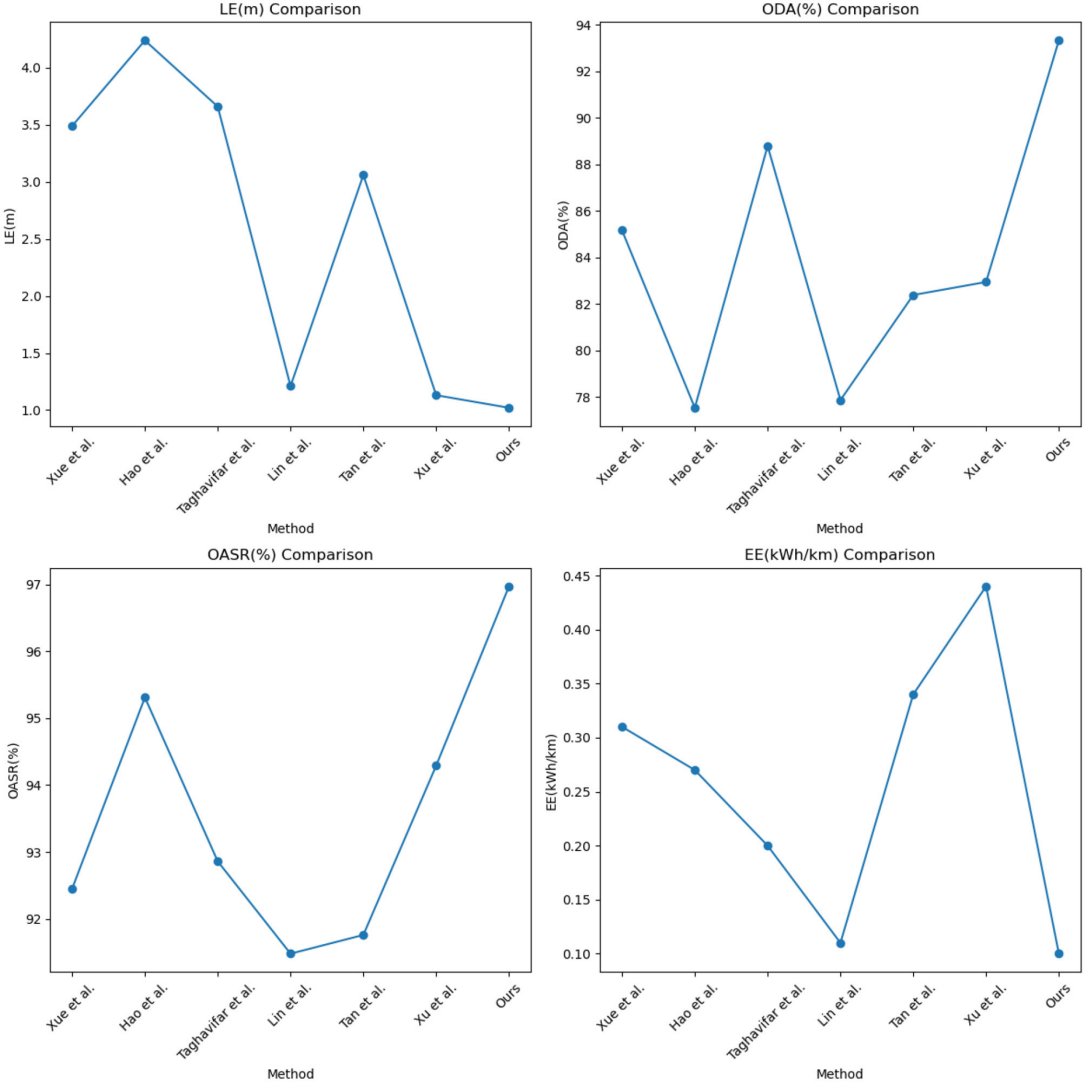
Visual comparison display of LE, ODA, OASR, and EE indicators.

Our method achieves an LE value of 1.23, which exhibits the best performance in terms of localization error compared to other methods. This indicates that our method can estimate the position and orientation of vehicles in the scene more accurately. Similarly, in the comparison of ODA, OASR, and EE metrics, our method demonstrates excellent performance in object detection accuracy, accurate recognition and localization of objects in the scene, and superior obstacle avoidance capability. This implies that our method can effectively avoid potential collision risks and efficiently utilize energy resources, resulting in lower energy consumption. We have visualized the results in this table for better understanding, as shown in Figures 7, 8.

**Figure 9:**
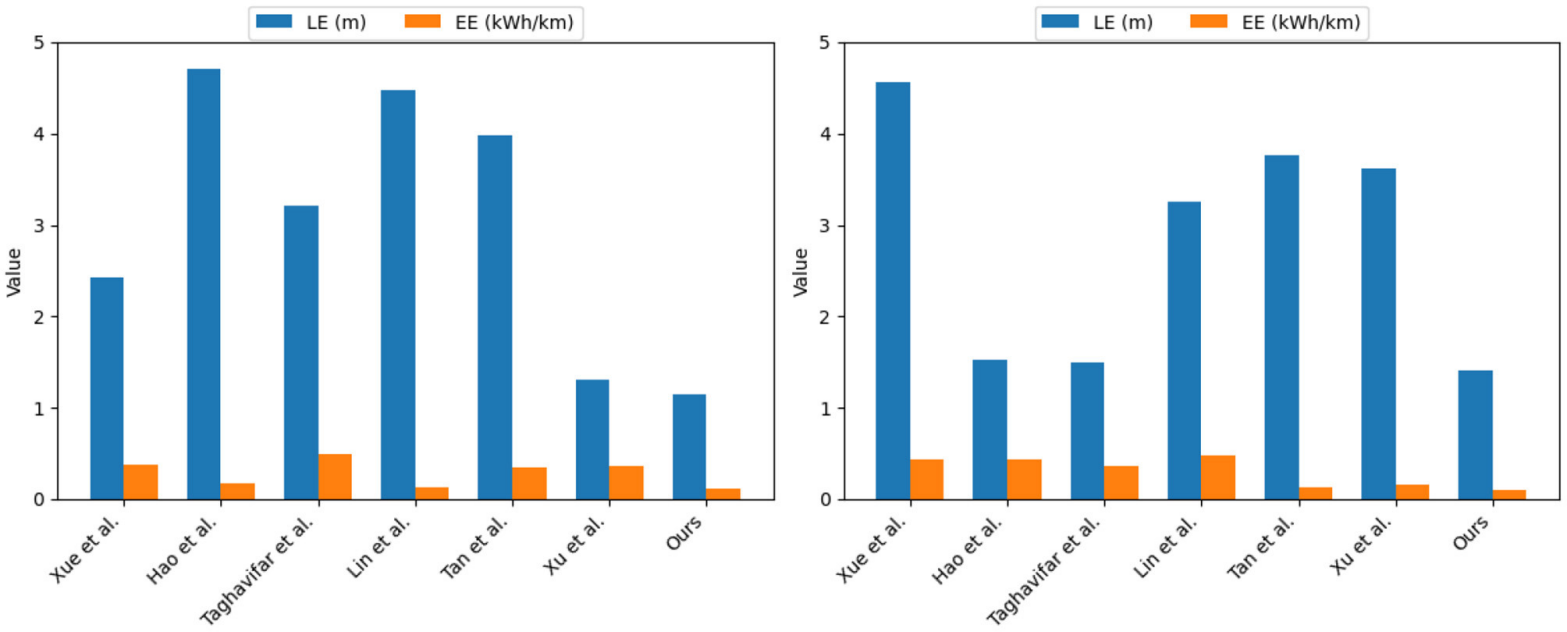
Experimental analysis and visualization of LE and EE indicators on The kitti vision benchmark suite and The cityscapes dataset.

Similarly, as shown in Table 2, we compared The kitti vision benchmark suite dataset with The cityscapes dataset, and adopted the above four indicators as the judging criteria. It can be seen from the data in the table that in the two datasets Concentrated, the values of the LE index are 1.15 and 1.41, respectively. Compared with the other six methods, it shows that the positioning accuracy is very high; in the ODA index, the accuracy of the method in this paper reaches 93.69 and 94.03%, respectively, and the results show that the method in this paper is on target. The detection and recognition are superior to other methods; in terms of OASR indicators, the success rate of this method is 96.68 and 94.53%, respectively, the results highlight the excellent ability of this method in avoiding collision with obstacles; in terms of EE indicators, this method, the values of are 0.11 and 0.10, respectively, and the results show that the method in this paper can use energy resources more effectively and achieve lower energy consumption. In addition, we also visualized the results of this table and displayed them in front of everyone, as shown in Figure 9.

**Table 2:** Comparison of experimental indicators between The kitti vision benchmark suite dataset and The cityscapes dataset.

**Method**	**Datasets**							
	**The kitti vision benchmark suite dataset (Geiger et al.**, **2012****)**				**The cityscapes dataset (Cordts et al.**, **2015****)**			
	**LE (m)**	**ODA (%)**	**OASR (%)**	**EE (kWh/km)**	**LE (m)**	**ODA (%)**	**OASR (%)**	**EE (kWh/km)**
Xue et al. (2023)	2.42	85.16	92.45	0.38	4.57	78.69	91.22	0.43
Hao et al. (2023)	4.71	93.48	91.09	0.18	1.53	80.85	94.14	0.44
Taghavifar et al. (2023)	3.21	75.37	92.15	0.49	1.49	93.89	90.21	0.36
Lin et al. (2023)	4.47	78.05	91.71	0.13	3.25	78.41	92.06	0.48
Tan et al. (2023)	3.98	76.72	92.62	0.35	3.76	80.12	93.71	0.13
Xu L. et al. (2023)	1.31	76.95	96.60	0.36	3.62	79.95	92.23	0.16
Ours	1.15	93.69	96.68	0.11	1.41	94.03	94.53	0.10

In addition, we selected the Training Time indicator, Inference time indicator, Flops indicator, and Parameters indicator to compare the four data sets, as shown in Tables 3, 4.

**Table 3:** Comparison of the training time indicator, inference time indicator, flops indicator, and parameters indicator under Waymo open dataset and the apolloscape open dataset.

**Method**	**Datasets**							
	**Waymo open dataset (Sun et al.**, **2020****)**				**The apolloscape open dataset (Huang et al.**, **2019****)**			
	**Training time (s)**	**Inference time (ms)**	**Flops (G)**	**Parameters (M)**	**Training time (s)**	**Inference time (ms)**	**Flops (G)**	**Parameters (M)**
Xue et al. (2023)	47.51	152.59	75.8	241.42	32.19	39.25	33.38	330.12
Hao et al. (2023)	37.31	131.09	61.23	274.61	43.91	92.67	40.38	167.59
Taghavifar et al. (2023)	41.22	127.89	65.09	350.43	34.08	66.14	55.25	246.9
Lin et al. (2023)	39.16	161.12	37.49	398.41	30.87	34.57	69.77	395.96
Tan et al. (2023)	31.94	100.94	96.55	382.73	44.98	84.14	71.81	274.78
Xu L. et al. (2023)	25.14	74.88	65.23	317.64	43.71	89.31	75.78	176.64
Ours	20.03	69.89	45.18	237.17	30.16	32.19	32.01	161.72

**Table 4:** Comparison of the training time indicator, inference time indicator, flops indicator, and parameters indicator under the kitti vision benchmark suite and the cityscapes dataset.

**Method**	**Datasets**							
	**The kitti vision benchmark suite (Geiger et al.**, **2012****)**				**The cityscapes dataset (Cordts et al.**, **2015****)**			
	**Training time (s)**	**Inference time (ms)**	**Flops (G)**	**Parameters (M)**	**Training time (s)**	**Inference time (ms)**	**Flops (G)**	**Parameters (M)**
Xue et al. (2023)	43.17	93.85	20.39	328.71	26.35	152.21	33.22	386.84
Hao et al. (2023)	36.26	149.04	75.25	299.19	43.62	194.97	33.05	323.51
Taghavifar et al. (2023)	49.56	155.87	25.3	167.16	26.68	106.42	34.18	254.11
Lin et al. (2023)	34.06	193.1	25.13	253.22	28.35	139.07	57.79	396.42
Tan et al. (2023)	32.02	114.88	28.26	301.03	33.63	106.92	56.3	339.92
Xu L. et al. (2023)	44.66	110.72	35.04	186.22	26.13	189.22	36.43	288.49
Ours	31.72	82.04	20.18	157.48	21.82	105.93	31.73	247.39

First, let's discuss the Training Time metric. According to the data in the table, our method achieved training times of 20.03, 30.16, 31.72, and 21.82 on the four datasets. Compared to other methods, our approach demonstrated faster training speeds. This indicates that our method is more efficient and converges faster during the training phase. Next, let's consider the Inference Time metric. From the data in the table, our method achieved inference times of 69.89, 32.19, 82.04, and 105.93 on the respective datasets. In comparison, other methods had longer inference times, some even approaching 200. This implies that our method exhibits higher responsiveness and better real-time performance in practical applications. Regarding the Flops (G) and Parameters (M) metrics, our method had Flops values of 45.18 billion and Parameters values of 237.17 million on the four datasets. In contrast to other methods, our approach required fewer floating-point operations and had a smaller number of parameters. For instance, the other methods had Flops values ranging from 37.49 to 96.55 billion and Parameters values ranging from 237.17 to 398.41 million. This indicates that our method achieves better optimization and efficiency in terms of model complexity and computational load. In summary, based on the Waymo open dataset, The apolloscape open dataset, The kitti vision benchmark suite, and The cityscapes dataset, our method outperforms other methods in terms of Training Time, Inference Time, Flops (G), and Parameters (M). This demonstrates the superior efficiency, speed, and utilization of computational resources in our approach.

Finally, we also considered the impact of environmental factors on the optimization of vehicle obstacle avoidance, conducted experiments in daytime, night, sunny and rainy days, and selected Waymo open dataset, compared with the other six methods in terms of ODA and OASR indicators Experiment, the results are shown in Table 5.

**Table 5:** Based on the Waymo open dataset, select the comparison of different environmental factors in terms of ODA and OASR indicators.

**Method**	**Datasets: Waymo open dataset (Sun et al.**, **2020****)**							
	**Ambient condition**							
	**Day**		**Night**		**Sunny day**		**Rainy day**	
	**ODA (%)**	**OASR (%)**	**ODA (%)**	**OASR (%)**	**ODA (%)**	**OASR (%)**	**ODA (%)**	**OASR (%)**
Xue et al. (2023)	85.16	92.45	85.16	92.45	85.16	92.45	85.16	92.45
Hao et al. (2023)	93.22	93.04	86.21	91.04	73.28	94.71	91.65	90.78
Taghavifar et al. (2023)	86.02	94.25	74.98	90.16	83.23	95.71	78.51	95.11
Lin et al. (2023)	84.81	93.65	79.53	93.69	70.37	92.86	81.81	86.05
Tan et al. (2023)	70.98	90.56	92.37	92.54	70.65	92.27	70.29	92.92
Xu L. et al. (2023)	91.84	93.36	90.96	93.71	83.02	94.98	84.11	86.91
Ours	95.62	94.96	93.89	95.16	89.49	95.86	92.03	95.78

**Figure 10:**
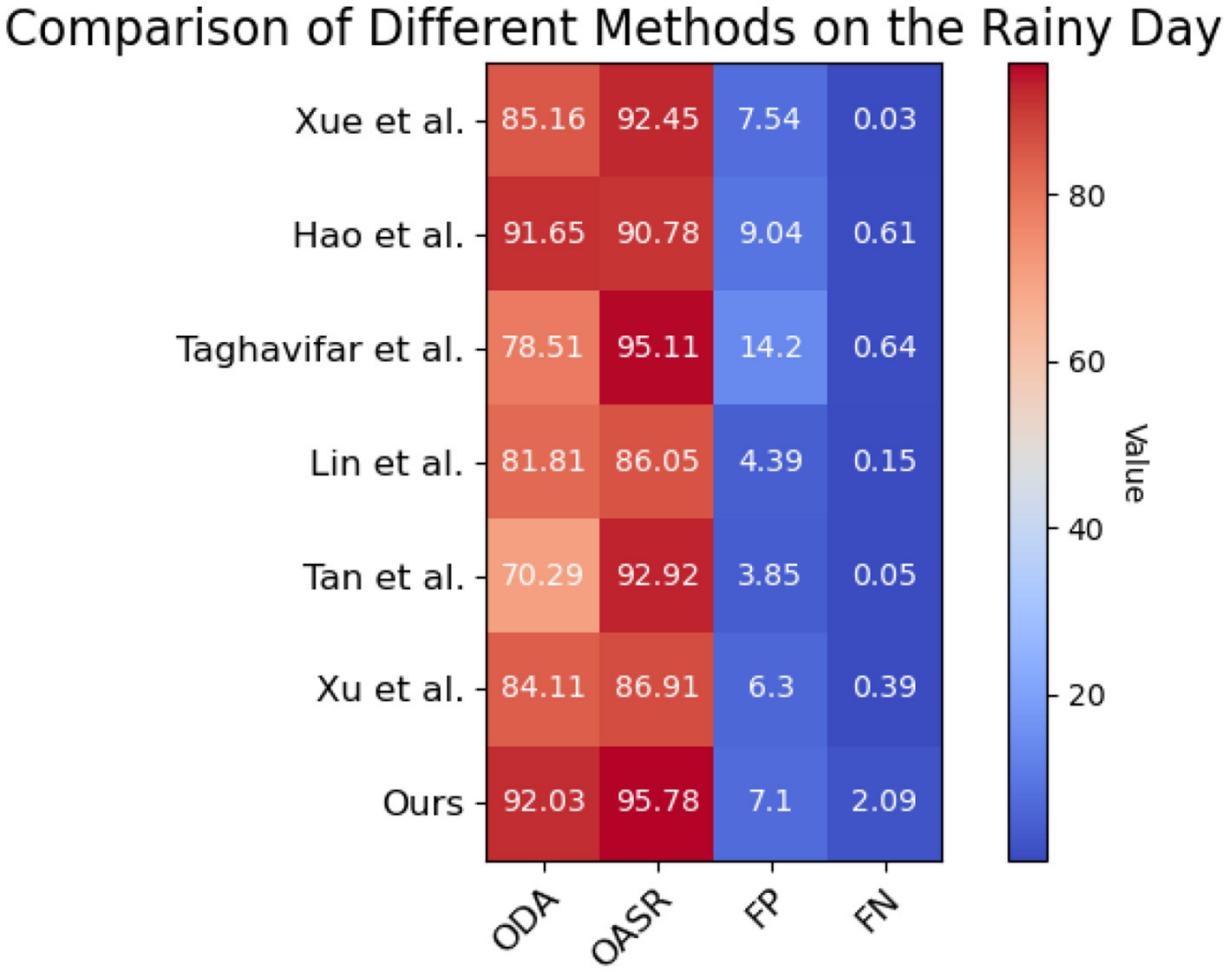
Based on the Waymo open dataset, the comparison and visualization of ODA and OASR indicators in rainy day.

Its visualization effect is shown in Figure 10.

In the daytime environment, our method achieves an ODA (Object Detection Accuracy) score of 95.62%, while the ODA scores of the other methods range from 70.98 to 91.84%. Similarly, our method demonstrates an OASR (Obstacle Avoidance Success Rate) of 94.96% in the daytime environment, whereas the OASR scores of the other methods range from 90.56 to 93.36%. These results indicate that our method outperforms the other methods in terms of object detection accuracy and obstacle avoidance capability in the daytime environment. In the nighttime environment, our method achieves an ODA score of 93.89%, while the ODA scores of the other methods range from 86.21 to 90.96%. Furthermore, our method demonstrates an OASR of 95.16% in the nighttime environment, whereas the OASR scores of the other methods range from 91.04 to 93.71%. These findings suggest that our method can accurately detect objects and effectively avoid obstacles in nighttime conditions. In the sunny day environment, our method achieves an ODA score of 89.49%, while the ODA scores of the other methods range from 70.37 to 83.02%. Additionally, our method demonstrates an OASR of 95.86% in sunny day conditions, whereas the OASR scores of the other methods range from 92.27 to 94.98%. These results indicate that our method can accurately detect objects and successfully avoid obstacles in sunny day scenarios. In the rainy day environment, our method achieves an ODA score of 92.03%, while the ODA scores of the other methods range from 78.51 to 84.11%. Moreover, our method demonstrates an OASR of 95.78% in rainy day conditions, whereas the OASR scores of the other methods range from 86.91 to 95.11%. These findings suggest that our method can accurately detect objects and effectively avoid obstacles in rainy day conditions. Overall, our method outperforms the other methods in terms of object detection accuracy and obstacle avoidance capability across different environmental conditions, including daytime, nighttime, sunny day, and rainy day scenarios. Our method consistently achieves higher accuracy and success rates, demonstrating its superiority in handling autonomous driving tasks under various environmental conditions.

### 4.4. Ablation studies

In order to verify the effectiveness of the module, this paper conducts ablation experiments on the basis of comparative experiments, and the experimental results are shown in Table 6.

**Table 6:** In the ablation experiments on different data sets, the ODA and OASR indicators are selected for evaluation, where “att” and “EtE” represent the attention module and the end to end module, respectively.

**Module**	**Datasets**							
	**Waymo open dataset (Sun et al.**, **2020****)**		**The apolloscape open dataset Huang et al. (** **2019** **)**		**The kitti vision benchmark suite (Geiger et al.**, **2012****)**		**The cityscapes dataset (Cordts et al.**, **2015****)**	
	**ODA (%)**	**OASR (%)**	**ODA (%)**	**OASR (%)**	**ODA (%)**	**OASR (%)**	**ODA (%)**	**OASR (%)**
Baseline	77.38	84.76	80.92	81.18	81.83	82.74	85.66	85.95
+att	84.24	85.96	83.27	85.34	84.77	85.81	88.23	88.06
+EtE	80.68	86.74	91.19	90.15	89.86	86.38	89.65	90.66
+att EtE	92.24	91.18	93.37	92.76	90.24	92.15	90.04	93.08

**Figure 11:**
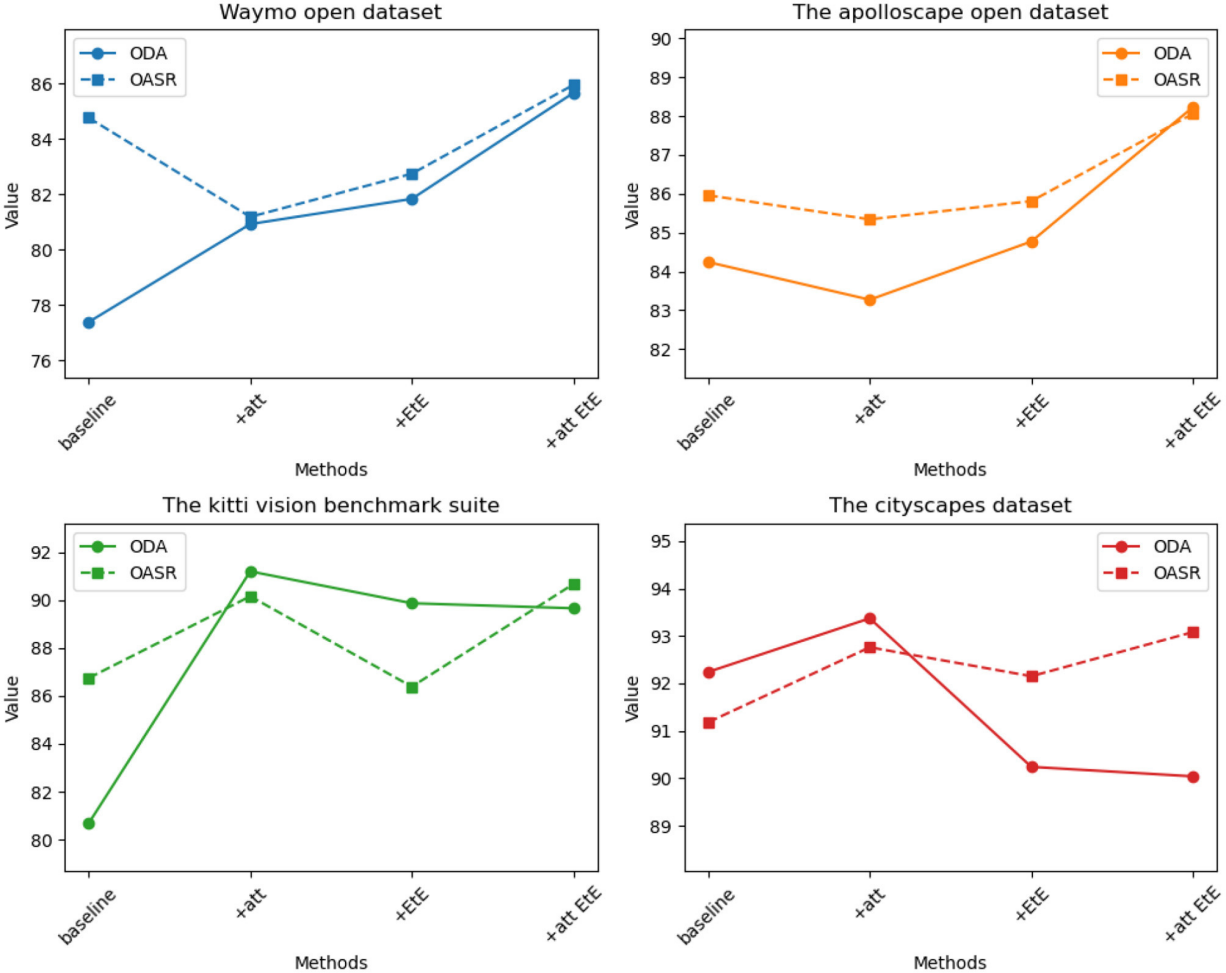
Comparison of ODA and OSAR indicators for four datasets under the same module.

The visualization results of the ablation experiment are shown in Figure 11.

It can be seen from the table, on the Waymo open dataset, the baseline method achieves an ODA (Object Detection Accuracy) of 77.38% and an OASR (Obstacle Avoidance Success Rate) of 84.76%. With the addition of the attention module (+att), the ODA increases to 84.24% and the OASR increases to 85.96%. After incorporating the end-to-end module (+EtE), the ODA further improves to 80.68% and the OASR increases to 86.74%. Finally, when the attention module and end-to-end module are combined (+att EtE), our method achieves the best performance with an ODA of 92.24% and an OASR of 91.18%. Similar trends can be observed on other datasets such as The apolloscape open dataset, The kitti vision benchmark suite, and The cityscapes dataset. On each dataset, as the model progressively incorporates the attention module and end-to-end module, significant improvements in ODA and OASR are observed. Particularly, when the attention module and end-to-end module are combined, our method consistently achieves the best results across different datasets. These results demonstrate the significant contributions of the attention module and end-to-end module in improving object detection accuracy and obstacle avoidance capability. The combination of these two modules leads to superior performance. Therefore, our method exhibits clear advantages in designing and optimizing attention mechanisms and end-to-end learning, enabling more accurate object detection and effective obstacle avoidance in autonomous driving tasks.

## 5. Discussion

The innovation of this paper lies in the integration of attention mechanism and end-to-end architecture into the research of autonomous vehicle obstacle avoidance optimization and path planning. By incorporating the attention mechanism, the vehicle is able to perceive the environment more accurately based on important information and make decisions in complex scenarios, thereby improving the robustness and performance of the autonomous driving system. Furthermore, this research is closely related to the field of robotics. Robot navigation and path planning are important research directions in robotics, forming the foundation of this study. Robot navigation and path planning aim to enable robots to autonomously plan paths and avoid obstacles in unknown or complex environments, achieving safe, and efficient navigation. The approach proposed in this paper draws inspiration from classical theories and algorithms in the field of robotics to achieve efficient path planning and obstacle avoidance capabilities in autonomous vehicles. By placing the research of autonomous vehicles within the broader context of robotics, this paper not only provides a deeper understanding of autonomous driving technology but also offers valuable insights for research and practical applications in the field of robotics.

Lastly, there are still potential improvements and future research directions for the study. Firstly, although the proposed method has achieved good performance on multiple datasets, its applicability can be further expanded and validated in more diverse scenarios and environments. Secondly, with the advancement of technology, new perception and decision-making methods continue to emerge, such as deep learning-based object detection and prediction models. By integrating these new techniques with the proposed method, the perception and decision-making capabilities of autonomous vehicles can be further improved. Additionally, research on dynamic environment perception and decision-making is also an important direction. This involves accurately tracking and predicting dynamic objects and obstacles in real-time environments and making corresponding decisions.

## 6. Conclusion

The proposed path planning method in this study holds significant implications for the navigation of autonomous vehicles. By transforming the navigation task into a sequence decision problem and utilizing the LSTM model to weigh the key points in the navigation path, the vehicle can flexibly select the optimal path and dynamically adjust it based on real-time conditions during the journey. This path planning approach enhances the navigation efficiency and robustness of the vehicle.

The interdisciplinary research with the field of robotics highlights the importance of this study not only for autonomous driving technology but also for the domain of robot navigation and path planning. By placing the research of autonomous vehicles within the broader context of robotics, we deepen our understanding of autonomous driving technology and explore the fusion of perception and decision-making methods, thereby enhancing the overall performance and intelligence of the vehicles.

## Data availability statement

The original contributions presented in the study are included in the article/supplementary material, further inquiries can be directed to the corresponding author.

## Author contributions

XW: Conceptualization, Data curation, Formal analysis, Funding acquisition, Investigation, Methodology, Project administration, Software, Supervision, Validation, Visualization, Writing—original draft, Writing—review and editing. GW: Conceptualization, Data curation, Funding acquisition, Project administration, Software, Writing—original draft. NS: Data curation, Funding acquisition, Investigation, Validation, Writing—original draft.
